# Take my breath away

**DOI:** 10.7554/eLife.25739

**Published:** 2017-04-04

**Authors:** Vinai C Thomas, Paul D Fey

**Affiliations:** 1 Center for Staphylococcal Research, Department of Pathology and Microbiology, University of Nebraska Medical Center, Omaha, United States; 1 Center for Staphylococcal Research, Department of Pathology and Microbiology, University of Nebraska Medical Center, Omaha, United Statespfey@unmc.edu

**Keywords:** Staphylococcus aureus,, respiration, biofilm, oxygen, cell death, cell lysis, Other

## Abstract

A lack of oxygen activates a pathway that causes the bacterial cell wall to break down, which, in turn, aids bacterial biofilm development.

**Related research article** Mashruwala AA, van de Guchte A, Boyd JM. 2017. Impaired respiration elicits SrrAB-dependent programmed cell lysis and biofilm formation in *Staphylococcus aureus*. *eLife* **6**:e23845. doi: 10.7554/eLife.23845

Although all bacteria are single-celled microorganisms, they display signs of multicellular behavior when they form communities known as biofilms. Within these communities, bacteria can sense cues from their environment, communicate with each other, differentiate into subpopulations, redistribute to form distinct architectures and cooperate in ways that enhance the ‘collective fitness’ of the biofilm.

At a molecular level, biofilm formation can be regarded as a complex developmental program that is controlled by multiple regulatory networks that effectively sense and respond to environmental signals (Moormeier and Bayles, 2017). Now, in eLife, Ameya Mashruwala, Adriana van de Guchte and Jeffrey Boyd of Rutgers University report how hypoxia (that is, a shortage of oxygen) influences the formation of biofilms in the bacterium *Staphylococcus aureus* (Mashruwala et al., 2017).

Staphylococcal biofilms are exposed to hypoxic conditions in a variety of environments, including when bacterial cell densities are high within the biofilm. A secreted sugar polymer called polysaccharide intercellular adhesin (PIA) is known to be involved in biofilm formation in some staphylococci, but very few clinical strains of *S. aureus* produce detectable levels of PIA in vitro. In fact, PIA is associated more with the protection of biofilms under conditions of high shear stress, rather than being a critical component of biofilm structure (Schaeffer et al., 2016; Foka et al., 2012; Weaver et al., 2012). Nevertheless, researchers have shown that hypoxia can increase PIA production, and thus biofilm formation, in strains that produce detectable levels of PIA (Cramton et al., 2001; Gotz, 2002).

Through a series of elegant experiments, Mashruwala et al. demonstrate that while hypoxia increases the biomass of the biofilm due to a decrease in cell respiration, the mechanism appears to be independent of PIA. Instead, they show that decreased respiration activates a pathway that results in cell lysis (that is, the breakdown of the cell wall that encloses the bacterium), which in turn leads to enhanced biofilm development.

Programmed cell death is thought to have evolved when multicellular organisms first originated. Since the individual cells that make up a multicellular organism are constrained for space and nutrients, they are limited in their capacity to multiply and must therefore cooperate to survive. Although a rather extreme form of cooperation, programmed cell death ensures that a multicellular body functions efficiently (Ameisen, 2002).

Similar to multicellular organisms, a bacterial biofilm may be viewed as a collection of cells that is confined within its extracellular matrix, which continuously controls the number of cells in the biofilm through regulated cell death and lysis mechanisms. This rather ruthless control of cell numbers has other benefits: for example, it increases the release of proteins and DNA from the bacteria, and these materials are then incorporated into the extracellular matrix in order to strengthen biofilm structure. Mashruwala et al. now demonstrate that hypoxia stimulates the release of proteins and DNA from the bacteria via cell lysis and that the depletion of extracellular protein and DNA (by protease or DNase treatments respectively) limits hypoxia mediated biofilm formation.

How does decreased respiration activate cell lysis? The critical component appears to be a signaling system called SrrAB, which is activated when an electron transporter called menaquinone remains chemically reduced. Once the SrrAB system has been activated, it increases the amount of the enzyme AtlA, a main player in cell destruction, and lowers the number of wall teichoic acids. In a healthy cell, the negative charge of the wall teichoic acids attracts protons and maintains an acidic microenvironment in the cell wall, which limits the activity and attachment of AtlA to the cell surface (Rice and Bayles, 2008; Biswas et al., 2012; Schlag et al., 2010). Mashruwala et al. also show that when the number of wall teichoic acids is low due to SrrAB activity, AtlA attaches more efficiently to the cell wall and is active enough to cause cell lysis.

How is this response ‘programmed’? In particular, is cell lysis an active response initiated by the cell following hypoxia, or is it a passive response due to an inability to adapt? The work of Mashruwala et al. clearly demonstrates that when various components of the programmed cell lysis pathway are inactive, cell lysis can be averted despite hypoxia. This suggests that cell lysis does not result from a general inability to adapt to hypoxia but rather is a bona fide programmed mechanism. However, outstanding questions remain. For instance, it is not clear why hypoxia only triggers lysis in some cells, despite all cells being genetically identical. Also, more research is needed to fully understand the relationship between cell lysis and programmed cell death during the development of a biofilm and how these processes overlap.
